# Supplementary material for: Content-rich biological network constructed by mining PubMed abstracts
Source: BMC Bioinformatics. 2004 Oct 8;5:147. doi: 10.1186/1471-2105-5-147 (PMC528731; doi:10.1186/1471-2105-5-147)
Supplement: Additional File 2 — The original results of the above study (non-essential files are deleted to keep the file size under the limit set by BMC bioinformatics). [file 1471-2105-5-147-S2.bz2 › chilibotAdditionalFile2/dip20/699ID7726836E18027/html/FTSZ_SULA.html]

 


 **FTSZ** and **SULA** 
  
Found 31 abstracts in PubMed, retrieved 20.  
 

 What does Google say? 
 PDF only 
| .edu only 

---

**Interactive relationship** (e.g. stimulation, inhibition, etc)

**Inhibitory relationship**- Bacterial SOS checkpoint protein  **SulA**  inhibits polymerization of purified  **FtsZ**  cell division protein.  Ref: 9683493 J Bacteriol, 1998
- **SulA**  prevented the formation of high molecular weight  **FtsZ**  polymers by interfering with  **FtsZ**  dimerization and subsequent oligomerization.  Ref: 10931335 Mol Microbiol, 2000
- This interaction was reduced by mutations in  **sulA**  and by most mutations in  **ftsZ**  that make cell refractory to  **sulA** .  Ref: 8752322 J Bacteriol, 1996
- Cell division inhibitors  **SulA**  and MinCD prevent formation of the  **FtsZ**  ring.  Ref: 8432706 J Bacteriol, 1993
- A cell division inhibitor  [ **SULA** ]   **SulA**  of Escherichia coli directly interacts with  **FtsZ**  through GTP hydrolysis.  Ref: 7726836 Biochem Biophys Res Commun, 1995
- Mutations in the essential cell division gene  **ftsZ**  confer resistance to  **SulA** , a cell division inhibitor  [ **SULA** ]  that is induced as part of the SOS response.  Ref: 8282688 J Bacteriol, 1994
- Reversal of  **SulA**  inhibition by blocking protein synthesis in  **SulA**  induced filaments led to a resumption of  **FtsZ**  ring formation and division.  Ref: 8432706 J Bacteriol, 1993
- The classic SOS gene product that inhibits cell division is SfiA or  **SulA** , which binds to  **FtsZ**  and prevents septum formation until the DNA damage has been repaired.  Ref: 9068638 J Bacteriol, 1997
- Instead,  **SulA**  binds the T7 loop surface of  **FtsZ** , opposite the nucleotide binding site, blocking polymer formation.  Ref: 12808143 Proc Natl Acad Sci U S A< MedlineTA>Proc Natl Acad Sci U S A, 1997
- **SulA**  protein inhibits the polymerization and the GTPase activity of  **FtsZ** , while point mutant  **SulA**  proteins show little effect on either of these  **FtsZ**  activities.  Ref: 9683493 J Bacteriol, 1998
- Furthermore, strains carrying various alleles of  **ftsZ** , selected on the basis of resistance to the inhibitor  **SulA** , displayed variable resistance to MinCD.  Ref: 11673433 J Bacteriol, 2001
- Whereas the  **ftsZ**  mutations were identical to a mutation known to produce a protein refractory to  **SulA**  inhibition, the  **sulA**  mutations affected the promoter operator region, reducing synthesis of  **SulA** .  Ref: 10368140 J Bacteriol, 1999
- It is proposed that when overproduced, the HslV HslU protease complex can degrade  **SulA**  which is an endogenous inhibitor of the essential cell division protein  **FtsZ**   [ **FTSZ** ] .  Ref: 9315728 FEBS Lett, 1997
- These results indicate that MinCD blocks the assembly of the septation machinery at a later step than  **SulA** , at the stage at which FtsA is added to the  **FtsZ**  ring.  Ref: 10931335 Mol Microbiol, 2000
- **SulA**  is induced in Escherichia coli by the SOS response and inhibits cell division through interaction with  **FtsZ** .  Ref: 9180687 Mol Gen Genet, 1997
**Stimulatory relationship**- These results indicate that  **SulA**  blocks Z ring formation by blocking  **FtsZ**  polymerization.  Ref: 9501185 Proc Natl Acad Sci U S A, 1998
- The revertants were found to carry a suppressor affecting either  **ftsZ**  encoding a key cell division protein or  **sulA**  encoding the  **SulA**  inhibitor, which binds and inhibits  **FtsZ** .  Ref: 10368140 J Bacteriol, 1999
- Here we show that a MalE  **SulA**  fusion that retains this inhibitory function in vivo inhibits the GTPase activity and polymerization of  **FtsZ**  in vitro.  Ref: 9501185 Proc Natl Acad Sci U S A, 1998
- Induction of the cell division inhibitor  [ **SULA** ]   **SulA** , a component of the SOS response, or the inhibitor MinCD, a component of the min system, blocked formation of the  **FtsZ**  ring and led to filamentation.  Ref: 8432706 J Bacteriol, 1993
**Neutral relationship**- An interaction was observed between  **FtsZ**  and  **SulA** , a component of the SOS response, and the interacting regions were mapped to their conserved domains.  Ref: 8752322 J Bacteriol, 1996
- It is likely that mutations in  **ftsZ**  that confer  **sulA**  resistance alter the conformation of the protein such that it assumes the active form.  Ref: 8282688 J Bacteriol, 1994
- Mutations in  **ftsZ**  that confer resistance to  **SulA**  affect the interaction of  **FtsZ**  with GTP.  Ref: 8282688 J Bacteriol, 1994
- The  **SulA**  protein interacts with the tubulin like  **FtsZ**  division protein.  Ref: 9683493 J Bacteriol, 1998
- Using purified  **SulA**  protein that was fused to the maltose binding protein, we demonstrate in vitro that  **SulA**  interacts with  **FtsZ**  to form a stable complex.  Ref: 7726836 Biochem Biophys Res Commun, 1995
- Addition of the  **SulA**  protein to the assay leads to rapid disassembly of existing  **FtsZ**  polymers, demonstrating its natural regulatory effect on  **FtsZ**  under the assay conditions.  Ref: 12202250 Anal Biochem, 2002
- **SulA**  halts cell division in Escherichia coli by binding to the major component of the division machinery  **FtsZ** .  Ref: 12808143 Proc Natl Acad Sci U S A< MedlineTA>Proc Natl Acad Sci U S A, 2002

**Non-interactive relationship** (e.g. studied together, co-existance, homology, etc.)

- We investigated the effects of purified  **SulA**  upon  **FtsZ** .  Ref: 9683493 J Bacteriol, 1998
- direct, reversible inhibition of  **FtsZ**  polymerization by  **SulA**  may account for division inhibition.  Ref: 9683493 J Bacteriol, 1998
- We have solved the crystal structure of  **SulA**  alone and in complex with  **FtsZ**  from Pseudomonas aeruginosa.  Ref: 12808143 Proc Natl Acad Sci U S A< MedlineTA>Proc Natl Acad Sci U S A, 1998
- **SulA**  inhibits cell division neither by binding the nucleotide binding site nor by inducing conformational changes in  **FtsZ** .  Ref: 12808143 Proc Natl Acad Sci U S A< MedlineTA>Proc Natl Acad Sci U S A, 1998
- MinC does not act like  **SulA** , which affects the GTPase activity of  **FtsZ** , and the model can explain how MinC would select for the  **FtsZ**  polymer rather than the monomer.  Ref: 11350934 EMBO J, 2001
- Crystal structure of the SOS cell division inhibitor  [ **SULA** ]   **SulA**  and in complex with  **FtsZ** .  Ref: 12808143 Proc Natl Acad Sci U S A< MedlineTA>Proc Natl Acad Sci U S A, 2001
- Inhibition of  **FtsZ**  polymerization by  **SulA** , an inhibitor of septation in Escherichia coli.  Ref: 9501185 Proc Natl Acad Sci U S A, 1998
- In this paper, size exclusion chromatography was used to study the effect of the  **SulA**  and MinCD division inhibitors on the oligomerization state of endogenous  **FtsZ**  in cytoplasmic extracts, and immunofluorescence microscopy was used to determine the effect of  **SulA**  and MinCD on the formation of  **FtsZ** , FtsA and ZipA rings at potential division sites.  Ref: 10931335 Mol Microbiol, 2000
- The block in initiation of division in the ftsW201 allele is shown to be independent of  **FtsZ**  or the  **FtsZ**  inhibitor,  **SulA** .  Ref: 7961485 J Bacteriol, 1994
- **SulA**  overproducing cells had a lysis positive phenotype, the ring structure but not the GTPase function of  **FtsZ**  was impaired.  Ref: 9732440 Arch Microbiol, 1998
